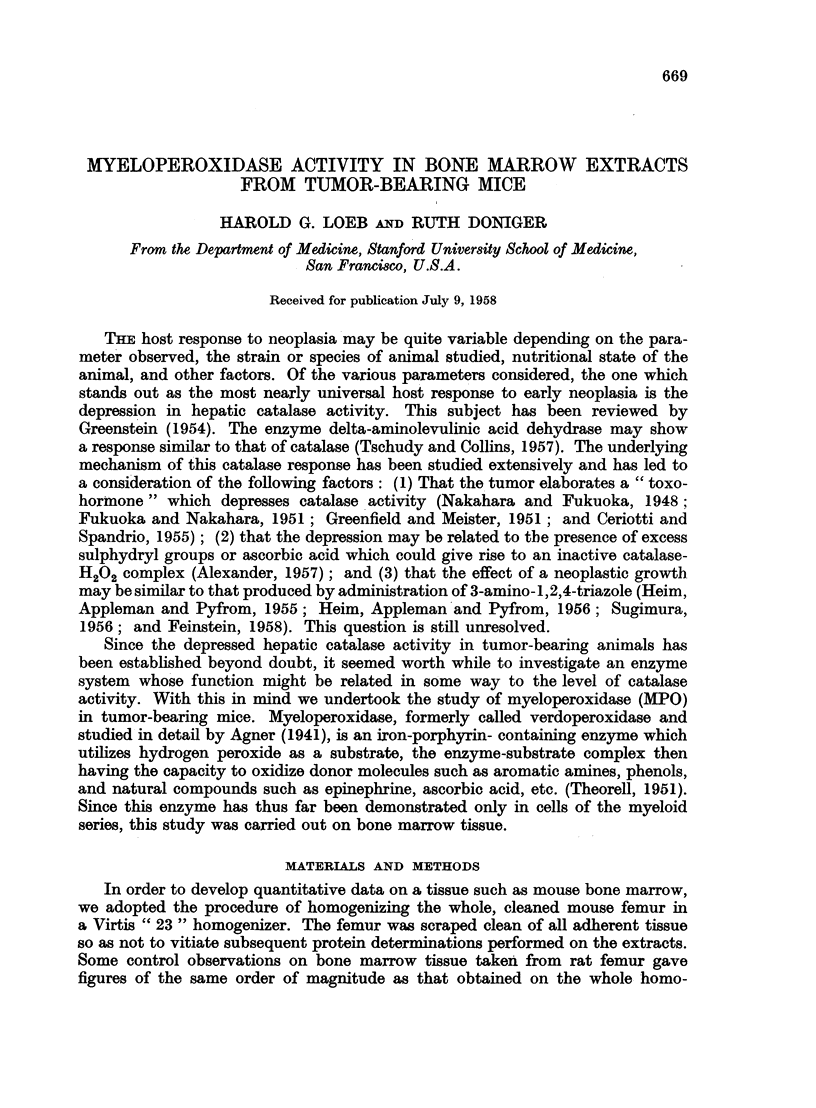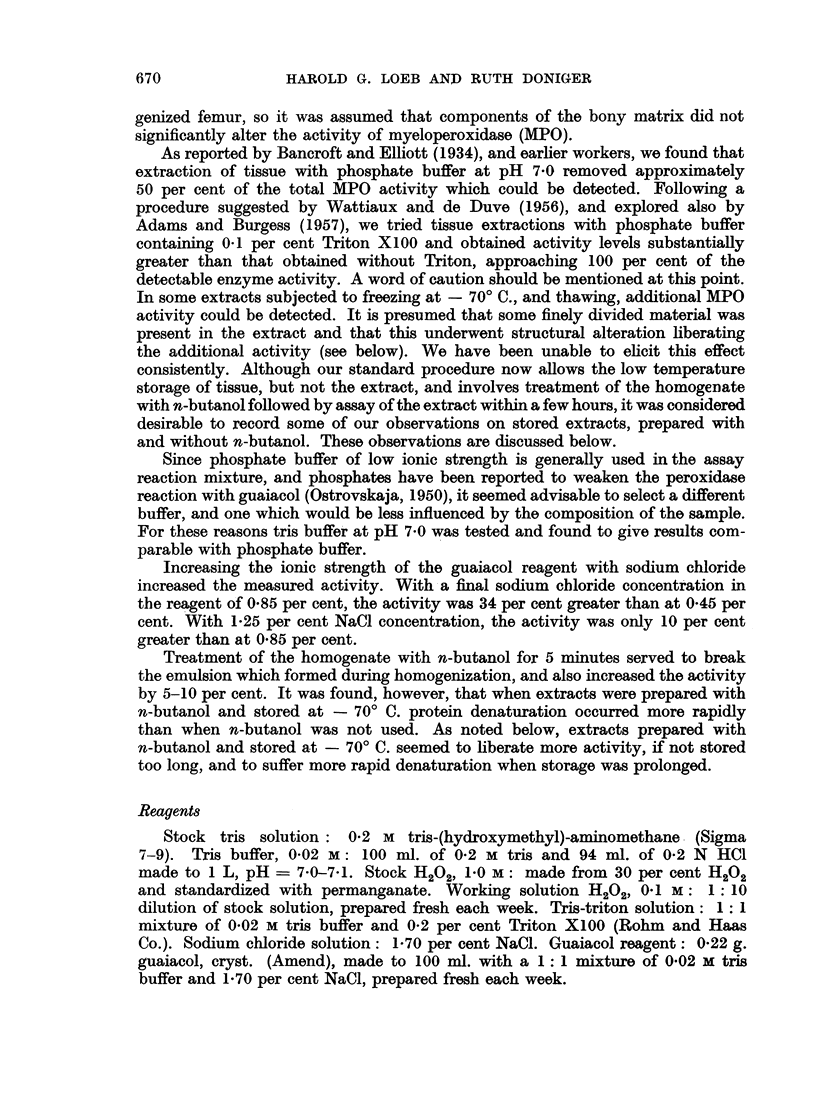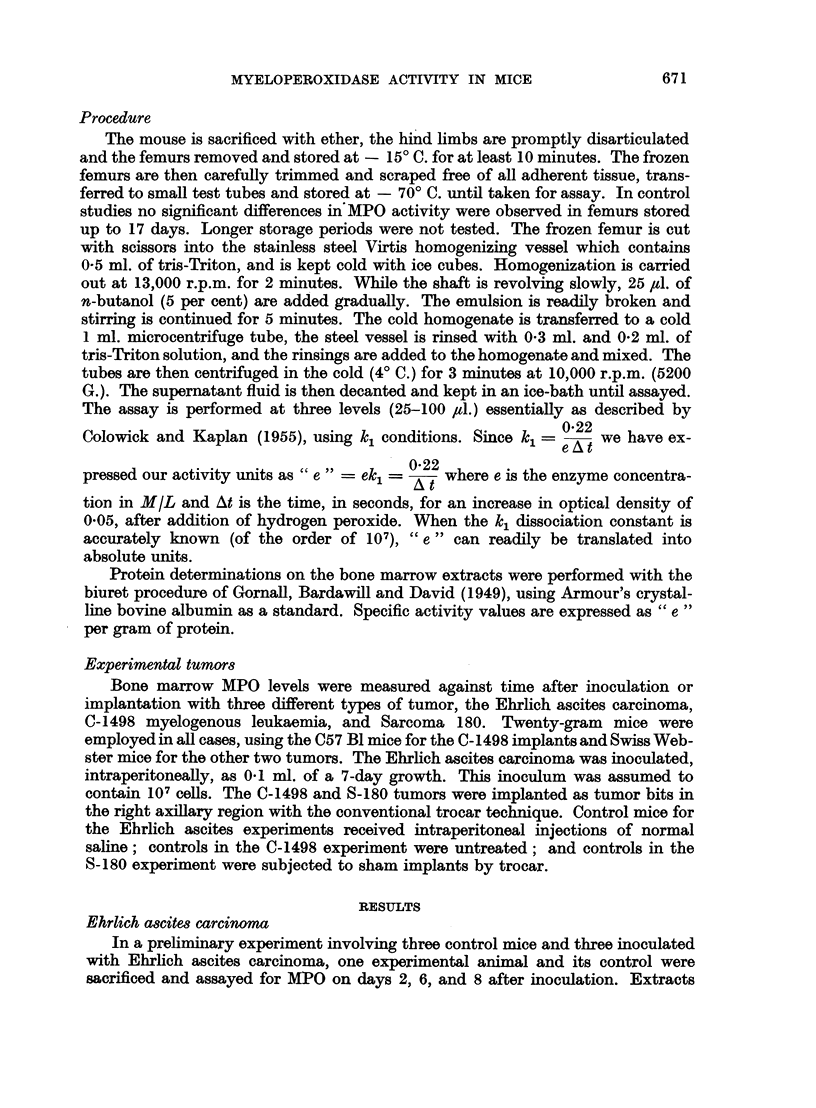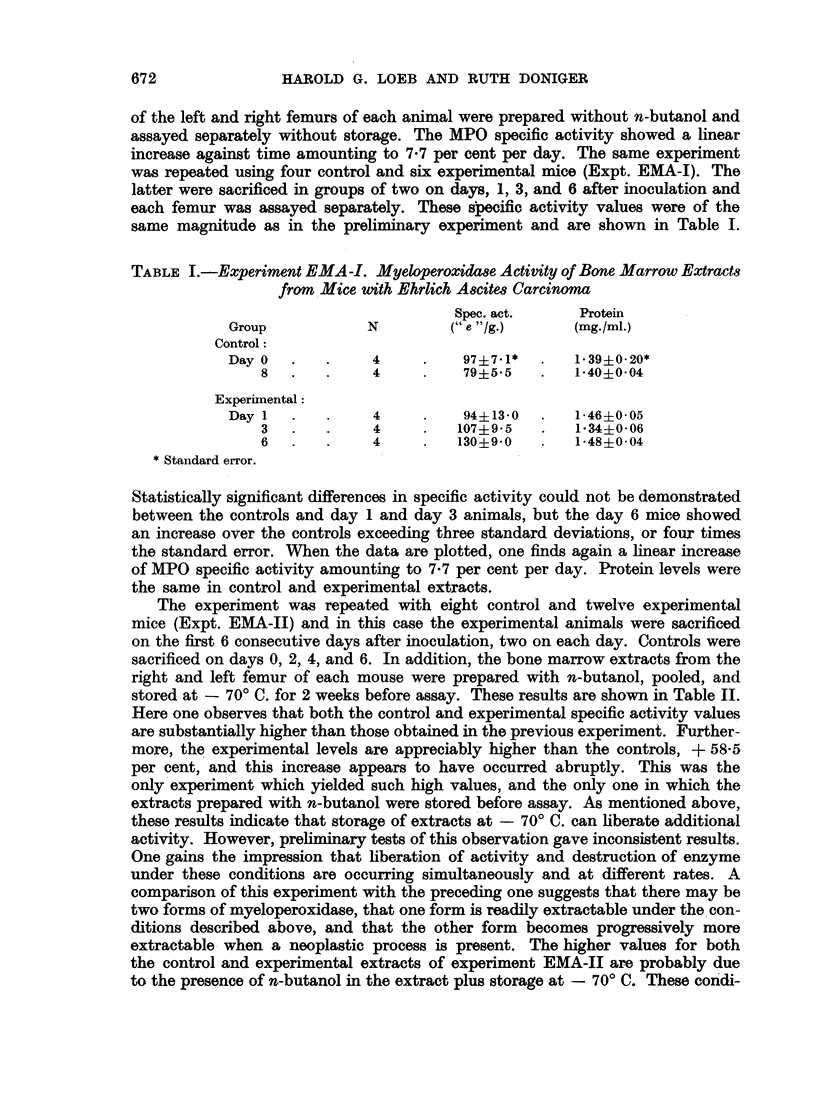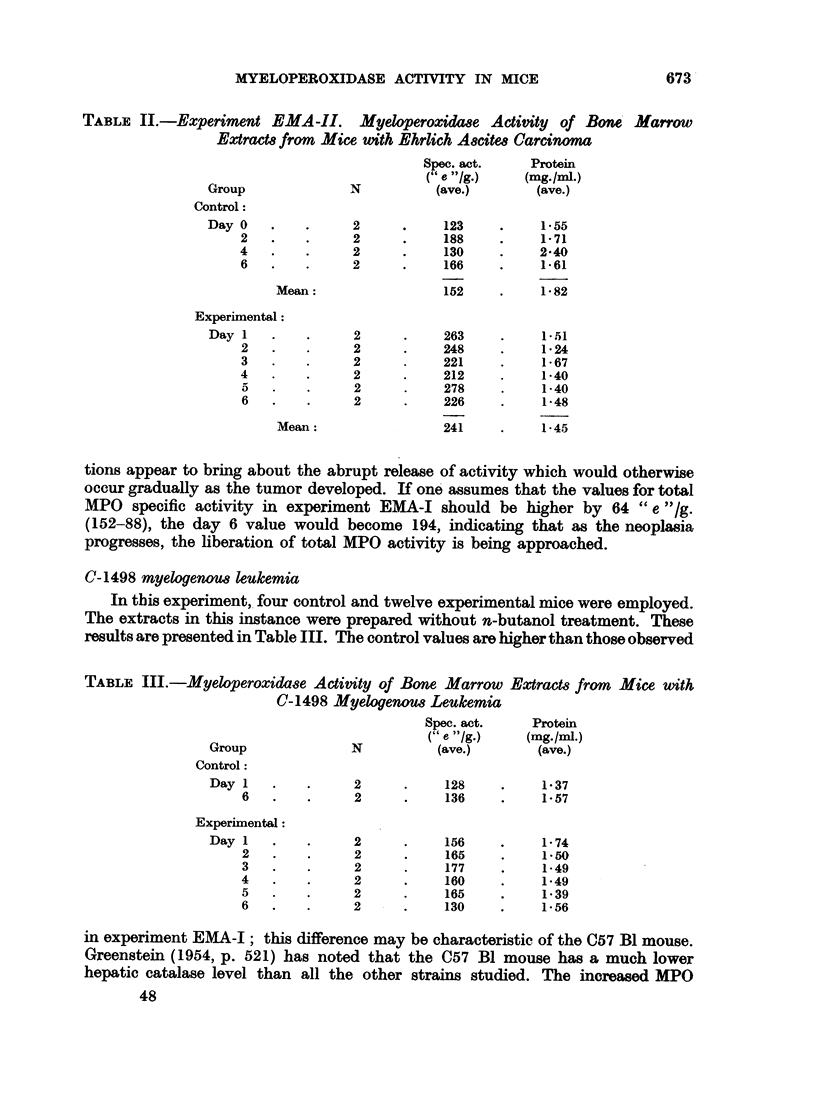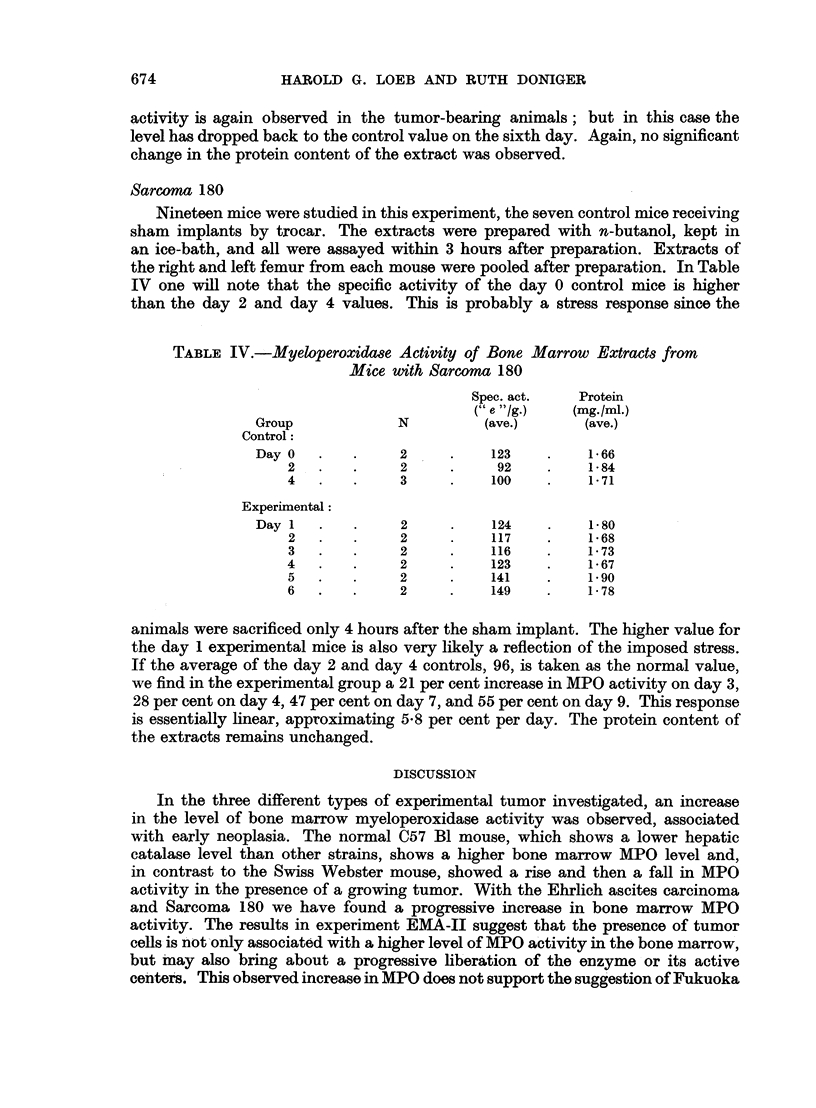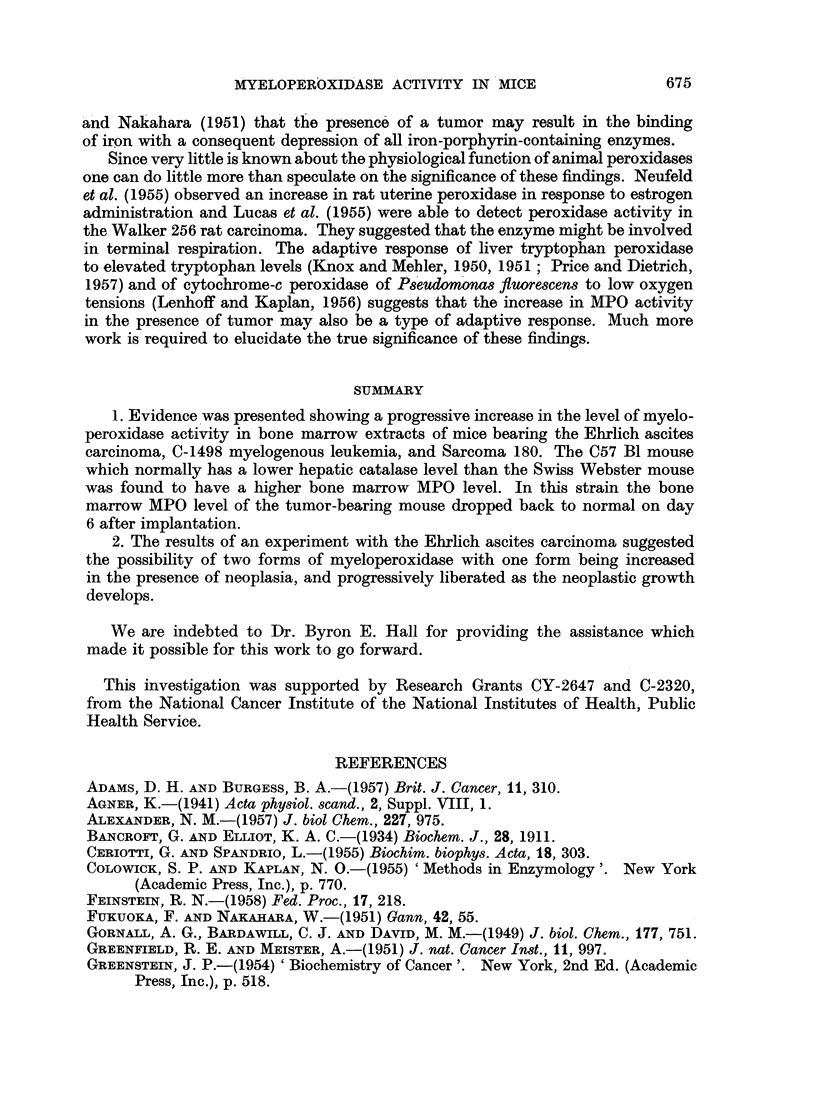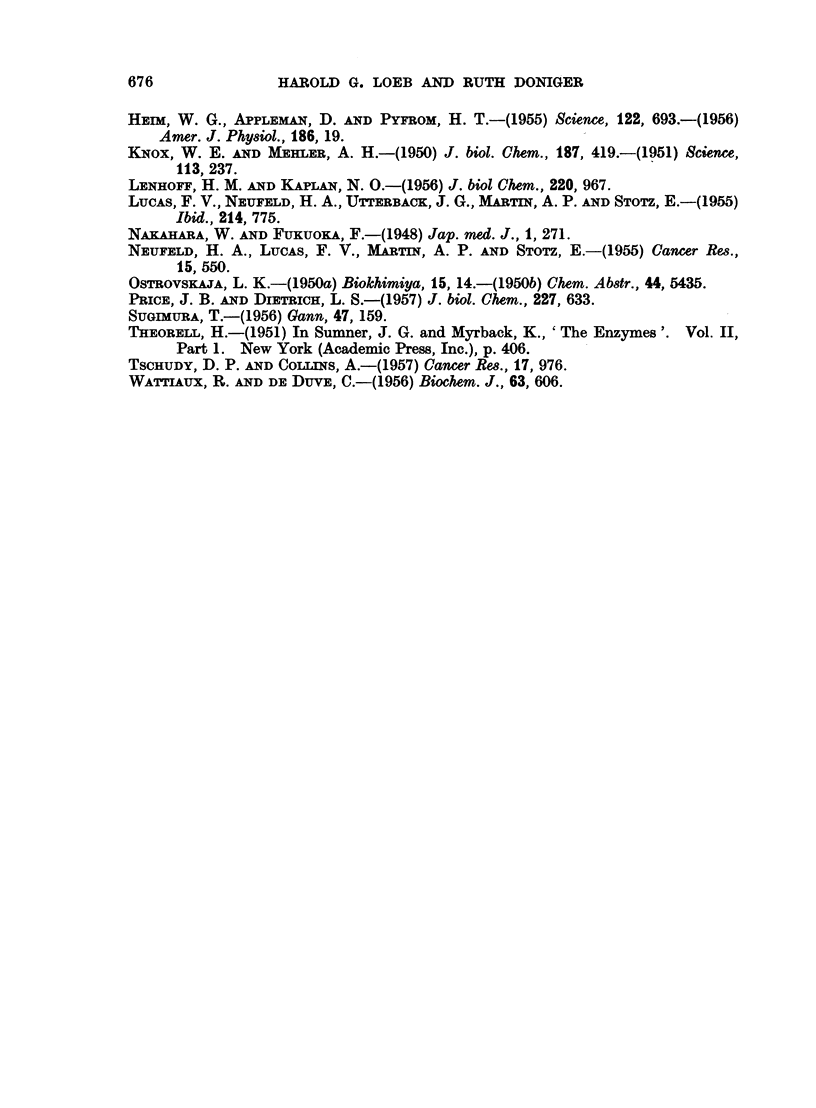# Myeloperoxidase Activity in Bone Marrow Extracts from Tumor-bearing Mice

**DOI:** 10.1038/bjc.1958.75

**Published:** 1958-12

**Authors:** Harold G. Loeb, Ruth Doniger


### MYELOPEROXIDASE ACTIVITY IN BONE MARROW EXTRACTS FROM TUMOR-BEARING MICE

HAROLD G. LOEB AND RUTH DONIGER

*From the Department of Medicine, Stanford University School of Medicine, San Francisco, U.S.A.*

Received for publication July 9, 1958

THE host response to neoplasia may be quite variable depending on the parameter observed, the strain or species of animal studied, nutritional state of the animal, and other factors. Of the various parameters considered, the one which stands out as the most nearly universal host response to early neoplasia is the depression in hepatic catalase activity. This subject has been reviewed by Greenstein (1954). The enzyme delta-aminolevulinic acid dehydrase may show a response similar to that of catalase (Tschudy and Collins, 1957). The underlying mechanism of this catalase response has been studied extensively and has led to a consideration of the following factors : (1) That the tumor elaborates a " toxohormone " which depresses catalase activity (Nakahara and Fukuoka, 1948 ; Fukuoka and Nakahara, 1951 ; Greenfield and Meister, 1951 ; and Ceriotti and Spandrio, 1955) ; (2) that the depression may be related to the presence of excess sulphydryl groups or ascorbic acid which could give rise to an inactive catalase-$H_2O_2$ complex (Alexander, 1957) ; and (3) that the effect of a neoplastic growth may be similar to that produced by administration of 3-amino-1,2,4-triazole (Heim, Appleman and Pyfrom, 1955 ; Heim, Appleman and Pyfrom, 1956 ; Sugimura, 1956 ; and Feinstein, 1958). This question is still unresolved.

Since the depressed hepatic catalase activity in tumor-bearing animals has been established beyond doubt, it seemed worth while to investigate an enzyme system whose function might be related in some way to the level of catalase activity. With this in mind we undertook the study of myeloperoxidase (MPO) in tumor-bearing mice. Myeloperoxidase, formerly called verdoperoxidase and studied in detail by Agner (1941), is an iron-porphyrin- containing enzyme which utilizes hydrogen peroxide as a substrate, the enzyme-substrate complex then having the capacity to oxidize donor molecules such as aromatic amines, phenols, and natural compounds such as epinephrine, ascorbic acid, etc. (Theorell, 1951). Since this enzyme has thus far been demonstrated only in cells of the myeloid series, this study was carried out on bone marrow tissue.

### MATERIALS AND METHODS

In order to develop quantitative data on a tissue such as mouse bone marrow, we adopted the procedure of homogenizing the whole, cleaned mouse femur in a Virtis " 23 " homogenizer. The femur was scraped clean of all adherent tissue so as not to vitiate subsequent protein determinations performed on the extracts. Some control observations on bone marrow tissue taken from rat femur gave figures of the same order of magnitude as that obtained on the whole homogenized femur, so it was assumed that components of the bony matrix did not significantly alter the activity of myeloperoxidase (MPO).

As reported by Bancroft and Elliott (1934), and earlier workers, we found that extraction of tissue with phosphate buffer at pH 7·0 removed approximately 50 per cent of the total MPO activity which could be detected. Following a procedure suggested by Wattiaux and de Duve (1956), and explored also by Adams and Burgess (1957), we tried tissue extractions with phosphate buffer containing 0·1 per cent Triton X100 and obtained activity levels substantially greater than that obtained without Triton, approaching 100 per cent of the detectable enzyme activity. A word of caution should be mentioned at this point. In some extracts subjected to freezing at — 70° C., and thawing, additional MPO activity could be detected. It is presumed that some finely divided material was present in the extract and that this underwent structural alteration liberating the additional activity (see below). We have been unable to elicit this effect consistently. Although our standard procedure now allows the low temperature storage of tissue, but not the extract, and involves treatment of the homogenate with *n*-butanol followed by assay of the extract within a few hours, it was considered desirable to record some of our observations on stored extracts, prepared with and without *n*-butanol. These observations are discussed below.

Since phosphate buffer of low ionic strength is generally used in the assay reaction mixture, and phosphates have been reported to weaken the peroxidase reaction with guaiacol (Ostrovskaja, 1950), it seemed advisable to select a different buffer, and one which would be less influenced by the composition of the sample. For these reasons tris buffer at pH 7·0 was tested and found to give results comparable with phosphate buffer.

Increasing the ionic strength of the guaiacol reagent with sodium chloride increased the measured activity. With a final sodium chloride concentration in the reagent of 0·85 per cent, the activity was 34 per cent greater than at 0·45 per cent. With 1·25 per cent NaCl concentration, the activity was only 10 per cent greater than at 0·85 per cent.

Treatment of the homogenate with *n*-butanol for 5 minutes served to break the emulsion which formed during homogenization, and also increased the activity by 5–10 per cent. It was found, however, that when extracts were prepared with *n*-butanol and stored at — 70° C. protein denaturation occurred more rapidly than when *n*-butanol was not used. As noted below, extracts prepared with *n*-butanol and stored at — 70° C. seemed to liberate more activity, if not stored too long, and to suffer more rapid denaturation when storage was prolonged.

*Reagents*

Stock tris solution : 0·2 M tris-(hydroxymethyl)-aminomethane (Sigma 7–9). Tris buffer, 0·02 M : 100 ml. of 0·2 M tris and 94 ml. of 0·2 N HCl made to 1 L, pH = 7·0–7·1. Stock $H_2O_2$, 1·0 M : made from 30 per cent $H_2O_2$ and standardized with permanganate. Working solution $H_2O_2$, 0·1 M : 1 : 10 dilution of stock solution, prepared fresh each week. Tris-triton solution : 1 : 1 mixture of 0·02 M tris buffer and 0·2 per cent Triton X100 (Rohm and Haas Co.). Sodium chloride solution : 1·70 per cent NaCl. Guaiacol reagent : 0·22 g. guaiacol, cryst. (Amend), made to 100 ml. with a 1 : 1 mixture of 0·02 M tris buffer and 1·70 per cent NaCl, prepared fresh each week.

*Procedure*

The mouse is sacrificed with ether, the hind limbs are promptly disarticulated and the femurs removed and stored at — 15° C. for at least 10 minutes. The frozen femurs are then carefully trimmed and scraped free of all adherent tissue, transferred to small test tubes and stored at — 70° C. until taken for assay. In control studies no significant differences in MPO activity were observed in femurs stored up to 17 days. Longer storage periods were not tested. The frozen femur is cut with scissors into the stainless steel Virtis homogenizing vessel which contains 0·5 ml. of tris-Triton, and is kept cold with ice cubes. Homogenization is carried out at 13,000 r.p.m. for 2 minutes. While the shaft is revolving slowly, 25 $\mu$l. of $n$-butanol (5 per cent) are added gradually. The emulsion is readily broken and stirring is continued for 5 minutes. The cold homogenate is transferred to a cold 1 ml. microcentrifuge tube, the steel vessel is rinsed with 0·3 ml. and 0·2 ml. of tris-Triton solution, and the rinsings are added to the homogenate and mixed. The tubes are then centrifuged in the cold (4° C.) for 3 minutes at 10,000 r.p.m. (5200 G.). The supernatant fluid is then decanted and kept in an ice-bath until assayed. The assay is performed at three levels (25–100 $\mu$l.) essentially as described by Colowick and Kaplan (1955), using $k_1$ conditions. Since $k_1 = \frac{0{\cdot}22}{e\,\Delta t}$ we have expressed our activity units as " $e$ " $= ek_1 = \frac{0{\cdot}22}{\Delta t}$ where $e$ is the enzyme concentration in $M/L$ and $\Delta t$ is the time, in seconds, for an increase in optical density of 0·05, after addition of hydrogen peroxide. When the $k_1$ dissociation constant is accurately known (of the order of $10^7$), " $e$ " can readily be translated into absolute units.

Protein determinations on the bone marrow extracts were performed with the biuret procedure of Gornall, Bardawill and David (1949), using Armour's crystalline bovine albumin as a standard. Specific activity values are expressed as " $e$ " per gram of protein.

*Experimental tumors*

Bone marrow MPO levels were measured against time after inoculation or implantation with three different types of tumor, the Ehrlich ascites carcinoma, C-1498 myelogenous leukaemia, and Sarcoma 180. Twenty-gram mice were employed in all cases, using the C57 Bl mice for the C-1498 implants and Swiss Webster mice for the other two tumors. The Ehrlich ascites carcinoma was inoculated, intraperitoneally, as 0·1 ml. of a 7-day growth. This inoculum was assumed to contain $10^7$ cells. The C-1498 and S-180 tumors were implanted as tumor bits in the right axillary region with the conventional trocar technique. Control mice for the Ehrlich ascites experiments received intraperitoneal injections of normal saline; controls in the C-1498 experiment were untreated; and controls in the S-180 experiment were subjected to sham implants by trocar.

### RESULTS

*Ehrlich ascites carcinoma*

In a preliminary experiment involving three control mice and three inoculated with Ehrlich ascites carcinoma, one experimental animal and its control were sacrificed and assayed for MPO on days 2, 6, and 8 after inoculation. Extracts of the left and right femurs of each animal were prepared without *n*-butanol and assayed separately without storage. The MPO specific activity showed a linear increase against time amounting to 7·7 per cent per day. The same experiment was repeated using four control and six experimental mice (Expt. EMA-I). The latter were sacrificed in groups of two on days, 1, 3, and 6 after inoculation and each femur was assayed separately. These specific activity values were of the same magnitude as in the preliminary experiment and are shown in Table I.

TABLE I.—*Experiment EMA-I. Myeloperoxidase Activity of Bone Marrow Extracts from Mice with Ehrlich Ascites Carcinoma*

| Group | N | Spec. act. ("*e*"/g.) | Protein (mg./ml.) |
|---|---|---|---|
| Control: | | | |
| Day 0 | 4 | 97±7·1* | 1·39±0·20* |
| 8 | 4 | 79±5·5 | 1·40±0·04 |
| Experimental: | | | |
| Day 1 | 4 | 94±13·0 | 1·46±0·05 |
| 3 | 4 | 107±9·5 | 1·34±0·06 |
| 6 | 4 | 130±9·0 | 1·48±0·04 |

* Standard error.

Statistically significant differences in specific activity could not be demonstrated between the controls and day 1 and day 3 animals, but the day 6 mice showed an increase over the controls exceeding three standard deviations, or four times the standard error. When the data are plotted, one finds again a linear increase of MPO specific activity amounting to 7·7 per cent per day. Protein levels were the same in control and experimental extracts.

The experiment was repeated with eight control and twelve experimental mice (Expt. EMA-II) and in this case the experimental animals were sacrificed on the first 6 consecutive days after inoculation, two on each day. Controls were sacrificed on days 0, 2, 4, and 6. In addition, the bone marrow extracts from the right and left femur of each mouse were prepared with *n*-butanol, pooled, and stored at − 70° C. for 2 weeks before assay. These results are shown in Table II. Here one observes that both the control and experimental specific activity values are substantially higher than those obtained in the previous experiment. Furthermore, the experimental levels are appreciably higher than the controls, + 58·5 per cent, and this increase appears to have occurred abruptly. This was the only experiment which yielded such high values, and the only one in which the extracts prepared with *n*-butanol were stored before assay. As mentioned above, these results indicate that storage of extracts at − 70° C. can liberate additional activity. However, preliminary tests of this observation gave inconsistent results. One gains the impression that liberation of activity and destruction of enzyme under these conditions are occurring simultaneously and at different rates. A comparison of this experiment with the preceding one suggests that there may be two forms of myeloperoxidase, that one form is readily extractable under the conditions described above, and that the other form becomes progressively more extractable when a neoplastic process is present. The higher values for both the control and experimental extracts of experiment EMA-II are probably due to the presence of *n*-butanol in the extract plus storage at − 70° C. These condi-

TABLE II.—*Experiment EMA-II. Myeloperoxidase Activity of Bone Marrow Extracts from Mice with Ehrlich Ascites Carcinoma*

| Group | N | Spec. act. ("e"/g.) (ave.) | Protein (mg./ml.) (ave.) |
|---|---|---|---|
| Control: | | | |
| Day 0 | 2 | 123 | 1·55 |
| 2 | 2 | 188 | 1·71 |
| 4 | 2 | 130 | 2·40 |
| 6 | 2 | 166 | 1·61 |
| Mean: | | 152 | 1·82 |
| Experimental: | | | |
| Day 1 | 2 | 263 | 1·51 |
| 2 | 2 | 248 | 1·24 |
| 3 | 2 | 221 | 1·67 |
| 4 | 2 | 212 | 1·40 |
| 5 | 2 | 278 | 1·40 |
| 6 | 2 | 226 | 1·48 |
| Mean: | | 241 | 1·45 |

tions appear to bring about the abrupt release of activity which would otherwise occur gradually as the tumor developed. If one assumes that the values for total MPO specific activity in experiment EMA-I should be higher by 64 "*e*"/g. (152–88), the day 6 value would become 194, indicating that as the neoplasia progresses, the liberation of total MPO activity is being approached.

*C*-1498 *myelogenous leukemia*

In this experiment, four control and twelve experimental mice were employed. The extracts in this instance were prepared without *n*-butanol treatment. These results are presented in Table III. The control values are higher than those observed

TABLE III.—*Myeloperoxidase Activity of Bone Marrow Extracts from Mice with C-1498 Myelogenous Leukemia*

| Group | N | Spec. act. ("e"/g.) (ave.) | Protein (mg./ml.) (ave.) |
|---|---|---|---|
| Control: | | | |
| Day 1 | 2 | 128 | 1·37 |
| 6 | 2 | 136 | 1·57 |
| Experimental: | | | |
| Day 1 | 2 | 156 | 1·74 |
| 2 | 2 | 165 | 1·50 |
| 3 | 2 | 177 | 1·49 |
| 4 | 2 | 160 | 1·49 |
| 5 | 2 | 165 | 1·39 |
| 6 | 2 | 130 | 1·56 |

in experiment EMA-I; this difference may be characteristic of the C57 Bl mouse. Greenstein (1954, p. 521) has noted that the C57 Bl mouse has a much lower hepatic catalase level than all the other strains studied. The increased MPO

activity is again observed in the tumor-bearing animals; but in this case the level has dropped back to the control value on the sixth day. Again, no significant change in the protein content of the extract was observed.

*Sarcoma* 180

Nineteen mice were studied in this experiment, the seven control mice receiving sham implants by trocar. The extracts were prepared with *n*-butanol, kept in an ice-bath, and all were assayed within 3 hours after preparation. Extracts of the right and left femur from each mouse were pooled after preparation. In Table IV one will note that the specific activity of the day 0 control mice is higher than the day 2 and day 4 values. This is probably a stress response since the

TABLE IV.—*Myeloperoxidase Activity of Bone Marrow Extracts from Mice with Sarcoma* 180

| Group | N | Spec. act. (" *e* "/g.) (ave.) | Protein (mg./ml.) (ave.) |
|---|---|---|---|
| Control: | | | |
| Day 0 | 2 | 123 | 1·66 |
| 2 | 2 | 92 | 1·84 |
| 4 | 3 | 100 | 1·71 |
| Experimental: | | | |
| Day 1 | 2 | 124 | 1·80 |
| 2 | 2 | 117 | 1·68 |
| 3 | 2 | 116 | 1·73 |
| 4 | 2 | 123 | 1·67 |
| 5 | 2 | 141 | 1·90 |
| 6 | 2 | 149 | 1·78 |

animals were sacrificed only 4 hours after the sham implant. The higher value for the day 1 experimental mice is also very likely a reflection of the imposed stress. If the average of the day 2 and day 4 controls, 96, is taken as the normal value, we find in the experimental group a 21 per cent increase in MPO activity on day 3, 28 per cent on day 4, 47 per cent on day 7, and 55 per cent on day 9. This response is essentially linear, approximating 5·8 per cent per day. The protein content of the extracts remains unchanged.

### DISCUSSION

In the three different types of experimental tumor investigated, an increase in the level of bone marrow myeloperoxidase activity was observed, associated with early neoplasia. The normal C57 Bl mouse, which shows a lower hepatic catalase level than other strains, shows a higher bone marrow MPO level and, in contrast to the Swiss Webster mouse, showed a rise and then a fall in MPO activity in the presence of a growing tumor. With the Ehrlich ascites carcinoma and Sarcoma 180 we have found a progressive increase in bone marrow MPO activity. The results in experiment EMA-II suggest that the presence of tumor cells is not only associated with a higher level of MPO activity in the bone marrow, but may also bring about a progressive liberation of the enzyme or its active centers. This observed increase in MPO does not support the suggestion of Fukuoka and Nakahara (1951) that the presence of a tumor may result in the binding of iron with a consequent depression of all iron-porphyrin-containing enzymes.

Since very little is known about the physiological function of animal peroxidases one can do little more than speculate on the significance of these findings. Neufeld *et al.* (1955) observed an increase in rat uterine peroxidase in response to estrogen administration and Lucas *et al.* (1955) were able to detect peroxidase activity in the Walker 256 rat carcinoma. They suggested that the enzyme might be involved in terminal respiration. The adaptive response of liver tryptophan peroxidase to elevated tryptophan levels (Knox and Mehler, 1950, 1951 ; Price and Dietrich, 1957) and of cytochrome-*c* peroxidase of *Pseudomonas fluorescens* to low oxygen tensions (Lenhoff and Kaplan, 1956) suggests that the increase in MPO activity in the presence of tumor may also be a type of adaptive response. Much more work is required to elucidate the true significance of these findings.

### SUMMARY

1. Evidence was presented showing a progressive increase in the level of myeloperoxidase activity in bone marrow extracts of mice bearing the Ehrlich ascites carcinoma, C-1498 myelogenous leukemia, and Sarcoma 180. The C57 Bl mouse which normally has a lower hepatic catalase level than the Swiss Webster mouse was found to have a higher bone marrow MPO level. In this strain the bone marrow MPO level of the tumor-bearing mouse dropped back to normal on day 6 after implantation.

2. The results of an experiment with the Ehrlich ascites carcinoma suggested the possibility of two forms of myeloperoxidase with one form being increased in the presence of neoplasia, and progressively liberated as the neoplastic growth develops.

We are indebted to Dr. Byron E. Hall for providing the assistance which made it possible for this work to go forward.

This investigation was supported by Research Grants CY-2647 and C-2320, from the National Cancer Institute of the National Institutes of Health, Public Health Service.

### REFERENCES

ADAMS, D. H. AND BURGESS, B. A.—(1957) *Brit. J. Cancer*, **11**, 310.
AGNER, K.—(1941) *Acta physiol. scand.*, **2**, Suppl. VIII, 1.
ALEXANDER, N. M.—(1957) *J. biol Chem.*, **227**, 975.
BANCROFT, G. AND ELLIOT, K. A. C.—(1934) *Biochem. J.*, **28**, 1911.
CERIOTTI, G. AND SPANDRIO, L.—(1955) *Biochim. biophys. Acta*, **18**, 303.
COLOWICK, S. P. AND KAPLAN, N. O.—(1955) ' Methods in Enzymology '. New York (Academic Press, Inc.), p. 770.
FEINSTEIN, R. N.—(1958) *Fed. Proc.*, **17**, 218.
FUKUOKA, F. AND NAKAHARA, W.—(1951) *Gann*, **42**, 55.
GORNALL, A. G., BARDAWILL, C. J. AND DAVID, M. M.—(1949) *J. biol. Chem.*, **177**, 751.
GREENFIELD, R. E. AND MEISTER, A.—(1951) *J. nat. Cancer Inst.*, **11**, 997.
GREENSTEIN, J. P.—(1954) ' Biochemistry of Cancer '. New York, 2nd Ed. (Academic Press, Inc.), p. 518.

HEIM, W. G., APPLEMAN, D. AND PYFROM, H. T.—(1955) *Science*, **122**, 693.—(1956) *Amer. J. Physiol.*, **186**, 19.
KNOX, W. E. AND MEHLER, A. H.—(1950) *J. biol. Chem.*, **187**, 419.—(1951) *Science*, **113**, 237.
LENHOFF, H. M. AND KAPLAN, N. O.—(1956) *J. biol Chem.*, **220**, 967.
LUCAS, F. V., NEUFELD, H. A., UTTERBACK, J. G., MARTIN, A. P. AND STOTZ, E.—(1955) *Ibid.*, **214**, 775.
NAKAHARA, W. AND FUKUOKA, F.—(1948) *Jap. med. J.*, **1**, 271.
NEUFELD, H. A., LUCAS, F. V., MARTIN, A. P. AND STOTZ, E.—(1955) *Cancer Res.*, **15**, 550.
OSTROVSKAJA, L. K.—(1950*a*) *Biokhimiya*, **15**, 14.—(1950*b*) *Chem. Abstr.*, **44**, 5435.
PRICE, J. B. AND DIETRICH, L. S.—(1957) *J. biol. Chem.*, **227**, 633.
SUGIMURA, T.—(1956) *Gann*, **47**, 159.
THEORELL, H.—(1951) In Sumner, J. G. and Myrback, K., ' The Enzymes '. Vol. II, Part 1. New York (Academic Press, Inc.), p. 406.
TSCHUDY, D. P. AND COLLINS, A.—(1957) *Cancer Res.*, **17**, 976.
WATTIAUX, R. AND DE DUVE, C.—(1956) *Biochem. J.*, **63**, 606.